# Characteristics of School-Associated Youth Homicides — United States, 1994–2018

**DOI:** 10.15585/mmwr.mm6803a1

**Published:** 2019-01-25

**Authors:** Kristin M. Holland, Jeffrey E. Hall, Jing Wang, Elizabeth M. Gaylor, Linda L. Johnson, Daniel Shelby, Thomas R. Simon, School-Associated Violent Deaths Study Group

**Affiliations:** ^1^Division of Violence Prevention, National Center for Injury Prevention and Control, CDC; ^2^Office of Minority Health and Health Equity, CDC; ^3^SciMetrika, LLC, McLean, Virginia.

To understand trends and characteristics in school-associated homicides involving youths, data from CDC’s School-Associated Violent Death Surveillance System were analyzed for 393 single-victim incidents that occurred during July 1994–June 2016 and 38 multiple-victim incidents (resulting in 121 youth homicides) during July 1994–June 2018. School-associated homicides consistently represent <2% of all youth homicides in the United States (*1*,*2*). The overall 22-year trend for single-victim homicide rates did not change significantly. However, multiple-victim incidence rates increased significantly from July 2009 to June 2018. Many school-associated homicides, particularly single-victim incidents, are similar to youth homicides unrelated to schools, often involving male, racial/ethnic minority youth victims, and occurring in urban settings. The majority of both single-victim (62.8%) and multiple-victim (95.0%) homicides were from a firearm-related injury. A comprehensive approach to violence prevention is needed to reduce risk for violence on and off school grounds.

The School-Associated Violent Death Surveillance System tracks lethal violence in school settings, providing a census of violent deaths (i.e., homicides, suicides, and legal intervention deaths) in school environments. Incidents are identified through a systematic media scan of computerized newspaper and broadcast media databases via LexisNexis (https://www.lexisnexis.com/) using keywords such as “shooting, death, violent, strangulation, beating, attack, stabbing, and died” combined with phrases including “primary or secondary or elementary or junior or high or middle or during or after or grounds or property or playground.” Cases include incidents where a fatality occurred 1) on a functioning public or private primary or secondary school campus in the United States; 2) while the victim was on the way to or from regular sessions at such a school; or 3) while the victim was attending or traveling to or from an official school-sponsored event. This study analyzed data for single-victim homicides during July 1994–June 2016 and multiple-victim incidents during July 1994–June 2018.*

Incidents involved the homicide of at least one youth^†^ aged 5–18 years, but could also include nonstudent (e.g., school staff members or family members) homicides. However, this study provides characteristics and trends for youth homicide victims only and does not include adult homicide victim data. Incidents identified through the media scan were confirmed with accounts from local law enforcement or school officials familiar with the incident, and law enforcement reports were collected when possible. Incident characteristics (e.g., victim and perpetrator demographics, school affiliation, victim-perpetrator relationship, incident location, cause and manner of death, and firearm information) during July 1994–June 2016 were coded from law enforcement reports or interviews with law enforcement or school officials familiar with each incident. When interviews or law enforcement reports were not obtained, data were abstracted from articles published in the media only when a reliable source (i.e., a law enforcement or school official, or judicial proceedings regarding the incident) was cited. Finally, eight multiple-victim incidents from July 2016 to June 2018 were identified through Internet and online database searches; data for these incidents were abstracted from media articles citing a reliable source.^§^ Overall, media reports were solely relied upon for coding demographic and circumstantial details for 80 (18.6%) of 431 incidents.

Victimization rates were calculated for school-aged youths involved in single- and multiple-victim incidents. Incidence rates (using the number of incidents as the numerator) were also calculated for multiple-victim incidents. Both rates used U.S. Department of Education and Current Population Survey^¶^ data on students enrolled in U.S. public and private primary and secondary schools by year as the denominator. National Center for Health Statistics mortality data for July 1994–June 2016 (the most recent school year for which data from both the School-Associated Violent Death Surveillance System and National Center for Health Statistics are available) served as the denominator for estimating the proportion of all homicides among school-age youths that were school-associated (*3*). School-associated homicide trends were analyzed using Joinpoint regression based on Poisson distribution. For multiple-victim homicide victimization rates, to account for dependence between cases, the variance was estimated by applying a compound Poisson process based on data aggregated across 3-year intervals (*4*).

During July 1994–June 2016, 423 school-associated homicide incidents occurred, including 393 (92.9%) single-victim and 30 (7.1%) multiple-victim incidents (accounting for 90 youth homicides), representing 1.2% of all homicides among youths aged 5–18 years (39,208) in the United States during this period. Further, three multiple-victim incidents occurred during July 2016–June 2017, and five occurred during July 2017–June 2018, accounting for 31 additional youth homicides.

Single-victim homicide decedents were mostly males (77.4%) and aged 15–18 years (77.9%), whereas multiple-victim decedents were evenly distributed among females (50.4%) and males (49.6%), and nearly a quarter were aged 5–9 years (Table 1). The single-victim homicide rate was highest in urban (0.07 per 100,000), public (0.037), and high schools (0.091), and was 8.27 times higher for non-Hispanic black youths than for non-Hispanic white youths (Table 2). Among those with known motives, gang-related activity (58.2%) and interpersonal disputes (44%) were the most common motives for single-victim homicides. Retaliation (e.g., due to bullying, rivalry between peer groups, or receiving bad grades from a teacher) (39.0%) was the most common motive for multiple-victim homicides, followed by gang-related activity (34.1%) and interpersonal disputes (29.3%). When single-victim incident perpetrators were known, the most common relationships between perpetrator and victim were stranger (27.6%), rival gang member (23.8%), or schoolmate/fellow student (21.2%). Multiple-victim homicide perpetrators were primarily strangers (36.2%) or schoolmates (36.2%) of their victims. Ninety-four (23.9%) single- and five (13.2%) multiple-victim incidents involved more than one perpetrator.

**TABLE 1:** School-associated youth homicide victim and perpetrator characteristics in single- (1994–2016) and multiple-victim (1994–2018) homicide incidents — United States, 1994–2018

Characteristic	July 1994–June 2016		July 1994–June 2018	
	Victims involved in single-victim incidents	Perpetrators involved in single-victim homicide incidents	Victims involved in multiple-victim homicide incidents*	Perpetrators involved in multiple-victim homicide incidents*
	No. (%)	No. (%)	No. (%)	No. (%)
**Total no. of victims or perpetrators**	**393**	**562**	**121**	**47**
**Sex**				
Male	304 (77.4)	452 (80.4)	60 (49.6)	46 (97.9)
Female	89 (22.6)	30 (5.3)	61 (50.4)	0 (—)
Unknown	0 (—)	80 (14.2)	0 (—)	1 (2.1)
**Race/Ethnicity**				
Black, non-Hispanic	208 (52.9)	218 (38.8)	15 (12.4)	11 (23.4)
White, non-Hispanic	92 (23.4)	68 (12.1)	84 (69.4)	22 (46.8)
Hispanic	38 (9.7)	89 (15.8)	8 (6.6)	8 (17.0)
Asian/Pacific Islander	10 (2.5)	22 (3.9)	4 (3.3)	0 (—)
American Indian/Alaska Native	1 (0.3)	2 (0.4)	7 (5.8)	2 (4.3)
Other	5 (1.3)	8 (1.4)	3 (2.5)	2 (4.3)
Unknown	39 (9.9)	155 (27.6)	0 (—)	2 (4.3)
**Age group (yrs)**				
5–9	12 (3.1)	1 (0.2)	28 (23.1)	0 (—)
10–14	75 (19.1)	37 (6.6)	28 (23.1)	6 (12.8)
15–18	306 (77.9)	277 (49.3)	65 (53.7)	23 (48.9)
19–24	0 (—)	101 (18.0)	0 (—)	9 (19.1)
≥25	0 (—)	20 (3.6)	0 (—)	8 (17.0)
Unknown	0 (—)	126 (22.4)	0 (—)	1 (2.1)
**Cause of death**				
Firearm	247 (62.8)	360 (64.1)	115 (95.0)	43 (91.5)
Stabbing	93 (23.7)	125 (22.2)	2 (1.7)	2 (4.3)
Blunt force	32 (8.1)	46 (8.2)	4 (3.3)	2 (4.3)
Asphyxiation	12 (3.1)	14 (2.5)	0 (—)	0 (—)
Other	8 (2.0)	16 (2.8)	0 (—)	0 (—)
Unknown	1 (0.3)	1 (0.2)	0 (—)	0 (—)
**Location of incident**				
On way to/from campus or school-sponsored event	200 (50.9)	304 (54.1)	10 (8.3)	7 (14.9)
On campus	186 (47.3)	246 (43.8)	111 (91.7)	40 (85.1)
At school-sponsored event	7 (1.8)	12 (2.1)	0 (—)	0 (—)
**Location of off-campus incident**				
Sidewalk/Path	91 (45.5)	147 (26.2)	5 (4.1)	3 (6.4)
Street	26 (13.0)	36 (6.4)	2 (1.7)	2 (4.3)
Parking lot	16 (8.0)	26 (4.6)	0 (—)	0 (—)
Bus stop	21 (10.5)	24 (4.3)	0 (—)	0 (—)
Motor vehicle	11 (5.5)	23 (4.1)	0 (—)	0 (—)
Athletic event/Field	6 (3.0)	10 (1.8)	0 (—)	0 (—)
On bus (school or public bus)	5 (2.5)	8 (1.4)	1 (0.8)	1 (2.1)
Playground/Park	3 (1.5)	5 (0.9)	0 (—)	0 (—)
Other	21 (10.5	25 (4.4)	2 (1.7)	1 (2.1)
**Location of on-campus incident**				
Parking lot	36 (19.4)	53 (9.4)	3 (2.5)	3 (6.4)
Campus lawn	25 (13.4)	37 (6.6)	5 (4.1)	2 (4.3)
Hallway	21 (11.3)	30 (5.3)	24 (19.8)	6 (12.8)
Athletic field/Court/Gymnasium	21 (11.3)	29 (5.2)	2 (1.7)	2 (4.3)
Sidewalk	16 (8.6)	19 (3.4)	1 (0.8)	0 (—)
School entrance	15 (8.1)	19 (3.4)	3 (2.5)	1 (2.1)
Bathroom	11 (5.9)	16 (2.8)	2 (1.7)	1 (2.1)
Playground	9 (4.8)	11 (2.0)	2 (1.7)	1 (2.1)
Cafeteria	7 (3.8)	7 (1.2)	10 (8.3)	4 (8.5)
Classroom/Library	7 (3.8)	7 (1.2)	51 (42.1)	10 (21.3)
Behind school building	3 (1.6)	4 (0.7)	3 (2.5)	7 (14.9)
Bus stop	3 (1.6)	3 (0.5)	0 (—)	0 (—)
Other	12 (6.5)	11 (2.0)	5 (4.1)	3 (6.4)
**School affiliation^†^**				
Student	361 (91.9)	250 (44.5)	112 (92.6)	18 (38.3)
No affiliation/Community resident	22 (5.6)	211 (37.5)	6 (5.0)	15 (31.9)
Student at another school	0 (—)	35 (6.2)	2 (1.7)	2 (4.3)
Former student	2 (0.5)	12 (2.1)	0 (—)	5 (10.6)
Student's parent/Guardian	0 (—)	5 (0.9)	0 (—)	2 (4.3)
Other relative of student	0 (—)	3 (0.5)	0 (—)	1 (2.1)
Faculty/Staff member	0 (—)	1 (0.2)	0 (—)	0 (—)
Other	0 (—)	0 (—)	0 (—)	1 (2.1)
Unknown	8 (2.0)	45 (8.0)	1 (0.8)	3 (6.4)
**Homicide-suicide**	10 (2.5)	10 (1.8)	51 (42.1)	10 (21.3)
**Relationship of perpetrator to victim^†^**				
Stranger	N/A	155 (27.6)	N/A	17 (36.2)
Rival gang member	N/A	134 (23.8)	N/A	1 (2.1)
Schoolmate/Fellow student	N/A	119 (21.2)	N/A	17 (36.2)
Residents of same community	N/A	47 (8.4)	N/A	3 (6.4)
Friend/Acquaintance	N/A	23 (4.1)	N/A	1 (2.1)
Dating partner	N/A	18 (3.2)	N/A	4 (8.5)
Relative	N/A	5 (0.9)	N/A	2 (4.3)
Faculty/Staff member	N/A	1 (0.2)	N/A	0 (—)
Unknown	N/A	60 (10.7)	N/A	2 (4.3)
**Motive^§,¶^**				
Gang-related activity	N/A	238 (58.2)	N/A	14 (34.1)
Interpersonal dispute	N/A	180 (44.0)	N/A	12 (29.3)
Brawl/Street fight	N/A	98 (24.0)	N/A	4 (9.8)
Retaliation	N/A	84 (20.5)	N/A	16 (39.0)
Dating partner problem/Lover's triangle	N/A	39 (9.5)	N/A	8 (19.5)
Sexual violence	N/A	20 (4.9)	N/A	8 (19.5)
Robbery	N/A	32 (7.8)	N/A	9 (22.0)
General characteristics^¶,^**				
Member of a gang	N/A	237 (56.3)	N/A	16 (38.1)
History of arrest	N/A	164 (39.0)	N/A	18 (42.9)
Regularly used alcohol/drugs	N/A	72 (17.1)	N/A	7 (16.7)
Intoxicated at time of incident	N/A	37 (8.8)	N/A	8 (19.0)
Mental health condition**				
Diagnosed	N/A	12 (2.9)	N/A	7 (16.7)
Suspected	N/A	8 (1.9)	N/A	7 (16.7)

**Abbreviation:** N/A = not applicable.

* Among 38 incidents.

^†^ School affiliation and victim-perpetrator relationship categories are mutually exclusive.

^§^ Information on motive was available for 409 (72.8%) single-victim and 41 (87.2%) multiple-victim homicide perpetrators. Percentages are based on the number of perpetrators with known motives.

^¶^ Motives and general characteristics are not mutually exclusive.

** General characteristics and mental health condition data were available for 421 (74.9%) single-victim and 42 (89.4%) multiple-victim homicide perpetrators. Percentages are based on the number of perpetrators with known characteristics.

**TABLE 2:** School-associated single- (1994–2016) and multiple-victim (1994–2017) homicide rates* among youths aged 5–18 years, by sex, race/ethnicity, and selected incident and school characteristics — United States, 1994–2017

Characteristic	July 1994–June 2016			July 1994–June 2017^†^			
	Single-victim homicide incidents (n = 393)			Multiple-victim homicide incidents (n = 33)			
	No. of youth deaths	Rate*	Rate ratio (95% CI)	No. of youth deaths	Rate*	Rate ratio (95% CI)	No. of incidents^§^
**All students**	393	0.0344	—	93	0.0078	—	33
**Sex^¶^**							
Female**	89	0.0176	—	47	0.0089	—	17
Male	304	0.0522	2.96 (2.34–3.75)	46	0.0076	0.85 (0.51–1.43)	21
**Race/Ethnicity^††^**							
White, non-Hispanic**	92	0.0144	—	62	0.0094	—	15
American Indian/Alaska Native	1	0.0081	0.56 (0.08–4.04)	7	0.0545	5.83 (1.10–30.89)	2
Asian	10	0.0199	1.38 (0.72–2.64)	1	0.0019	0.2 (0.05–0.85)	1
Black, non-Hispanic	208	0.1195	8.27 (6.47–10.57)	13	0.0071	0.76 (0.31–1.91)	11
Hispanic	38	0.0175	1.21 (0.83–1.77)	7	0.0030	0.33 (0.11–0.93)	5
Other/Unknown^§§^	44	—	—	3	––	—	6
**Fatal firearm injury**							
No**	145	0.0127	—	6	0.0005	—	3
Yes	247	0.0216	1.7 (1.39–2.09)	87	0.0073	14.5 (4.04–52.03)	30
Unknown^§§^	1	—	—	0	––	—	0
**School locale^††^**							
Rural**	61	0.0177	—	22	0.0061	—	9
Suburban	98	0.0239	1.35 (0.98–1.86)	54	0.0125	2.05 (0.61–6.90)	10
Urban	234	0.0702	3.97 (2.99–5.26)	17	0.0049	0.8 (0.31–2.07)	14
**School type^¶^**							
Private**	11	0.0103	—	5	0.0045	—	1
Public	380	0.0369	3.57 (1.96–6.50)	88	0.0082	1.81 (0.23–13.94)	32
Unknown^§§^	2	—	—	0	––	—	0
**School level^¶^**							
Elementary/Middle**	108	0.0136	—	41	0.0049	—	14
High/Combination	285	0.0908	6.67 (5.34–8.32)	52	0.0157	3.18 (0.99–10.19)	19

**Abbreviation:** CI = confidence interval.

* Per 100,000 students enrolled in U.S. public and private primary and secondary schools.

^†^ National Center for Education Statistics data were available only through the 2016–17 school year. Data from the 2017–18 school year are not yet available; thus, rate calculations for multiple-victim homicides (n = 93) are for the period from 1994–95 through 2016–17.

^§^ Number of incidents for sex and race/ethnicity categories do not sum to 33 because incidents involved several possible combinations of these variables (e.g., one incident could involve only males, only females, or a combination of both males and females).

^¶^ U.S. Current Population Survey data on all students enrolled in U.S. schools by sex, school type, and school level were used for denominators. Calculations using Current Population Survey data were limited to students who were aged 5–18 years and enrolled in grades K–12.

** Referent category.

^††^ National Center for Education Statistics Common Core of Data, Public School Universe Survey, Private School Universe Survey, and State Nonfiscal Public Elementary/Secondary Education Survey were used to identify the number of students enrolled in U.S. public and private schools by race/ethnicity and school locale for rate calculation denominators. These calculations were limited to grades K–12.

^§§^ Rate and rate ratio were not calculated for “Unknown.”

Firearm injuries were the cause of death in 247 (62.8%) single-victim school-associated homicides and 35 (92.1%) multiple-victim incidents that resulted in 115 (95%) youth homicides (Supplementary Table, https://stacks.cdc.gov/view/cdc/61748). Among these, more than one firearm was used in 10 (4.0%) single-victim and five (14.3%) multiple-victim incidents. In addition, 40.0% of single-victim and 60.5% of multiple-victim homicide perpetrators who used firearms were aged <18 years.

Overall, the average rate of single-victim school-associated youth homicides during July 1994–June 2016 was 0.03 per 100,000 students, and the average rate of multiple-victim school-associated homicides during July 1994–June 2017 was 0.008 per 100,000 (Table 2). Single-victim homicide rates increased significantly from July 2000 to June 2007 after a decline since July 1994; however, the rate did not change significantly over the entire period (p = 0.3) (Figure 1). Multiple-victim homicide victimization rates fluctuated substantially annually, but did not indicate a significant trend for the overall period (p = 0.6) (Figure 2). However, multiple-victim homicide incidence rates declined during July 1994–June 2009 and then increased through June 2018. Incidence rates fluctuated substantially (range = 0–6 incidents per year), and the recent increase likely was related to eight incidents that occurred during July 2016–June 2018.

**FIGURE 1:**
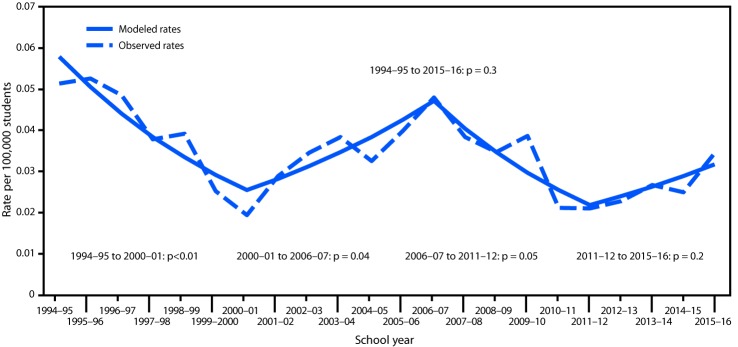
Victimization rates* for school-associated single-victim homicides per 100,000 students — United States, July 1994–June 2016 * Victimization rates were calculated as number of school-aged youth victims (i.e., aged 5–18 years) as the numerator and number of students enrolled in U.S. primary and secondary public and private schools as denominators. For single-victim school-associated homicides, incidence rates are equivalent to victimization rates. Single-victim homicide trends were analyzed using Joinpoint regression based on Poisson distribution, and the predicted rates from the model are shown as modeled rates.

**FIGURE 2:**
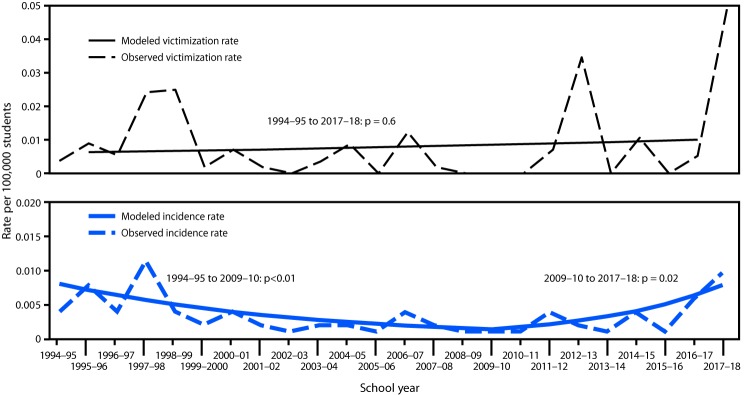
Victimization* and incidence rates^†^ of school-associated multiple-victim homicide per 100,000 students — United States, July 1994–June 2018^§^ * Victimization rates were calculated as number of school-aged youth victims (i.e., aged 5–18 years) as the numerator and number of students enrolled in U.S. primary and secondary public and private schools as the denominator. ^†^ Incidence rates were calculated as number of school-associated youth homicide incidents as the numerator and number of students enrolled in U.S. primary and secondary public and private schools as the denominator. ^§^ School-associated homicide trends were analyzed using Joinpoint regression based on Poisson distribution. For victimization rates, to account for dependence between multiple-victim cases, the variance was estimated by applying a compound Poisson process based on data aggregated across 3-year intervals. The predicted rates from the model are shown as modeled rates.

## Discussion

Although school-associated youth homicides account for <2% of all youth homicides, they are devastating for families, schools, and entire communities; lessons learned from studying these incidents can have broad implications for youth violence prevention. Approximately 90% of school-associated homicide incidents during July 1994–June 2016 involved only one victim. The remaining incidents during this time frame involved multiple victims and accounted for a substantial number of decedents (90; 18.6% of all youth victims during this time). Single-victim school-associated homicide rates did not change significantly overall despite fluctuations over time. Conversely, multiple-victim school-associated homicide incidence decreased from July 1994 to June 2009, but then increased significantly through the 2017–18 school year. These results, highlighting the proportion of youth homicides that are school-associated and the fluctuation in annual trends, are consistent with previous research (*1*,*2*).

Single-victim school-associated homicide characteristics are consistent with national data indicating that racial/ethnic minority adolescents are at higher risk for being homicide victims than are non-Hispanic white youths, and that youth homicide rates are higher in urban areas (*5*,*6*). The frequent connections with gang activity and interpersonal disputes suggest that school-associated homicides might often be a reflection of broader community-wide risks (*7*). Firearm-related injuries were the cause of death for 70.4% of all youth school-associated homicides included in this study. Further, many perpetrators of firearm-involved incidents were aged <18 years. Research has shown that most firearms used by youths in school-associated violent death incidents were obtained from their own home or from a friend or relative, underscoring the need to ensure safe storage and to restrict minors’ unsupervised access to firearms (*8*).

The findings in this report are subject to at least five limitations. First, only incidents reported in the media are included in this study, and changes in media coverage could affect trends. It is possible that some incidents could have been missed; however, incident data were compared with other online data sources (e.g., Gun Violence Archive [https://www.gunviolencearchive.org/]) containing information on school-associated homicides to ensure that the surveillance system captured cases described elsewhere if they met inclusion criteria. Second, circumstantial data collected through interviews were susceptible to recall bias, given that interviews were conducted after incidents occurred. Third, only multiple-victim incidents were included in analyses for the 2 most recent school years. Therefore, the single-victim trend analysis ended in June 2016. It is unlikely that multiple-victim incidents would be omitted in the case identification process for the 2 most recent school years, given the extensive media coverage that such incidents garner. Fourth, while only media reports citing reliable sources were used when law enforcement reports were unavailable, the information in these reports might be subject to reporting biases and might not be comprehensive in nature. Finally, statistical power for some comparisons was limited because of small numbers.

The number of school-associated youth homicides remains unacceptably high. The findings indicating that the characteristics of many school-associated homicides resemble youth homicides in the broader community suggest the need for prevention beyond the school setting. CDC’s *A Comprehensive Technical Package for the Prevention of Youth Violence and Associated Risk Behaviors* can help states, communities, and schools implement approaches based on the best available evidence (*9*). For example, communities experiencing gang and firearm violence might benefit from street outreach programs (e.g., Cure Violence, Safe Streets) that train persons with credibility in the community (e.g., former gang members) to change community norms and reduce escalating conflicts. CDC’s technical package describes a range of prevention options, including strategies that promote connections between youths and caring adults, enhance youths’ problem-solving and coping skills, and reduce risk among youths who have been violent. A comprehensive approach could address risk for youth violence on and off school property.

**Summary**

**What is already known about this topic?**

Patterns in single- and multiple-victim school-associated homicide rates differ, and both fluctuate annually.

**What is added by this report?**

Single-victim homicide rates remained stable overall during 1994–2016. School-associated single-victim homicides share characteristics with youth homicides in the community, often involving racial/ethnic minorities, males aged 15–18 years, and occurring in urban areas. Firearm-related injuries were the cause of death in 247 (62.8%) and 115 (95%) single- and multiple-victim homicides, respectively. Multiple-victim incidence rates increased significantly from July 2009 to June 2018.

**What are the implications for public health practice?**

Evidence-based youth violence prevention options exist, including strategies that promote connections between youths and caring adults, enhance problem-solving and coping skills, and reduce risk among youths who have been violent. A comprehensive violence prevention approach is important for reducing violence on and off school grounds.

## Footnotes

* Approximately 36,000 news articles per school year are reviewed in the media scan to identify an average of 50 potential single-victim cases annually. The validation of cases and access to incident data creates data lags. Manual Internet searches using phrases such as “school shooting” and “multiple victims and school” and web-based firearm injury data sets (i.e., Everytown for Gun Safety and the Gun Violence Archive) enhance the validation process and facilitate access to incident data for multiple-victim incidents. The overall process resulted in data currently being available for single-victim incidents through June 2016 and multiple victim incidents through July 2018.

† Although all incidents in this study involved school-aged youths, some youths might not have been enrolled as students in a school.

§ 2016–17 and 2017–18 multiple-victim cases were identified through manual Internet searches using phrases such as “school shooting” and “multiple victims and school,” as well as supplementary review of web-based firearm injury data sets (i.e., Everytown for Gun Safety and the Gun Violence Archive) to identify cases matching the School-Associated Violent Death Surveillance System case definition for multiple-victim youth homicides. Demographic and circumstance data for these cases were abstracted from media sources that referenced reliable sources as described above.

¶ National Center for Education Statistics Common Core of Data, Public School Universe Survey, Private School Universe Survey, and State Nonfiscal Public Elementary/Secondary Education Survey were used to identify the number of students enrolled in U.S. public and private schools by race/ethnicity and school locale for rate calculation denominators. These calculations were limited to grades K–12. National Center for Education Statistics data were available only through the 2016–17 school year. Data from the 2017–18 school year are not yet available; thus, rate calculations for multiple-victim homicides (n = 93) are for the period from 1994–95 through 2016–17. U.S. Current Population Survey data on all students enrolled in U.S. schools by sex, school type, school level, and school year were used for denominators. Calculations using Current Population Survey data were limited to students aged 5–18 years and enrolled in grades K–12.
